# Successful revascularization and minimal intestinal reconstruction of a secondary aortoduodenal fistula

**DOI:** 10.1007/s12328-026-02320-5

**Published:** 2026-04-01

**Authors:** Yoko Azuma, Rumi Itoyama, Yuki Kitano, Shigeki Nakagawa, Takashi Yoshinaga, Hirohisa Okabe, Hiromitsu Hayashi, Toshihiro Fukui, Masaaki Iwatsuki

**Affiliations:** 1 Department of Gastroenterological Surgery, Graduate School of Life Sciences, Kumamoto University, 1-1-1 Honjo, Kumamoto, 860-8556 Japan; 2 Department of Cardiovascular Surgery, Graduate School of Life Sciences, Kumamoto University, 1-1-1 Honjo, Kumamoto, 860-8556 Japan

**Keywords:** Secondary aortoenteric fistula (SAEF), Revascularization, Intestinal reconstruction

## Abstract

**Introduction:**

Secondary aortoenteric fistula (SAEF) is a rare complication following artificial vascular replacement. We present a patient who underwent successful surgical treatment for SAEF > 40 years after the initial surgery.

**Case presentation:**

A 66-year-old woman underwent right subclavian artery–abdominal aorta bypass surgery for abdominal aortic stenosis due to Takayasu disease 43 years earlier. Endoscopy revealed exposure of the artificial vascular graft in the descending duodenum. Bilateral axillary artery–femoral artery artificial vascular bypass surgery was performed prior to removing the original artificial vessels. However, hypotension developed intraoperatively. Therefore, the artificial vascular graft was partially resected, and another bypass surgery was performed with a new artificial vessel between the original artificial vessels and the femoral artery. The duodenum was then resected as a partial wedge, with side-to-side anastomosis with the jejunum.

**Conclusion:**

In this case, SAEF was diagnosed by endoscopy and treated with successful revascularization and minimal intestinal reconstruction.

## Introduction

Secondary aortoenteric fistula (SAEF) is a relatively rare complication that occurs in 0.3%–1.6% of patients after abdominal aortic replacement surgery. The main causes are pseudoaneurysms at the anastomosis of the artificial vessel and mechanical irritation of the intestinal tract by the artificial vessel [1]. Although numerous treatments have been proposed, there is no established treatment [2]; thus, the choice of treatment options is often difficult.

In this report, we discuss a patient who had undergone artificial vessel replacement > 40 years earlier, and was subsequently treated successfully with surgery for SAEF.

## Case presentation

**Fig. 1:**
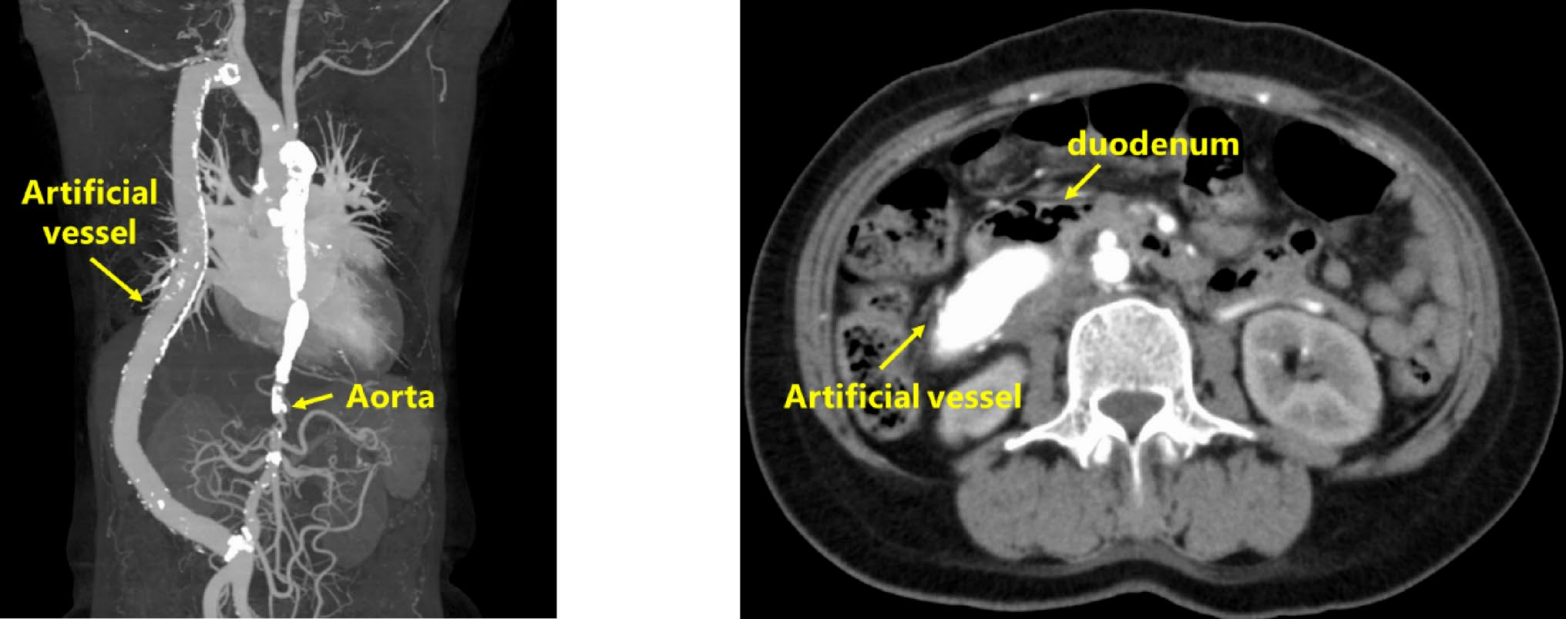
Computed tomography (CT) findings. CT images showing the artificial blood vessel positioned near the duodenum and compressing the descending duodenum, with no obvious active bleeding. CT, computed tomography

**Fig. 2:**
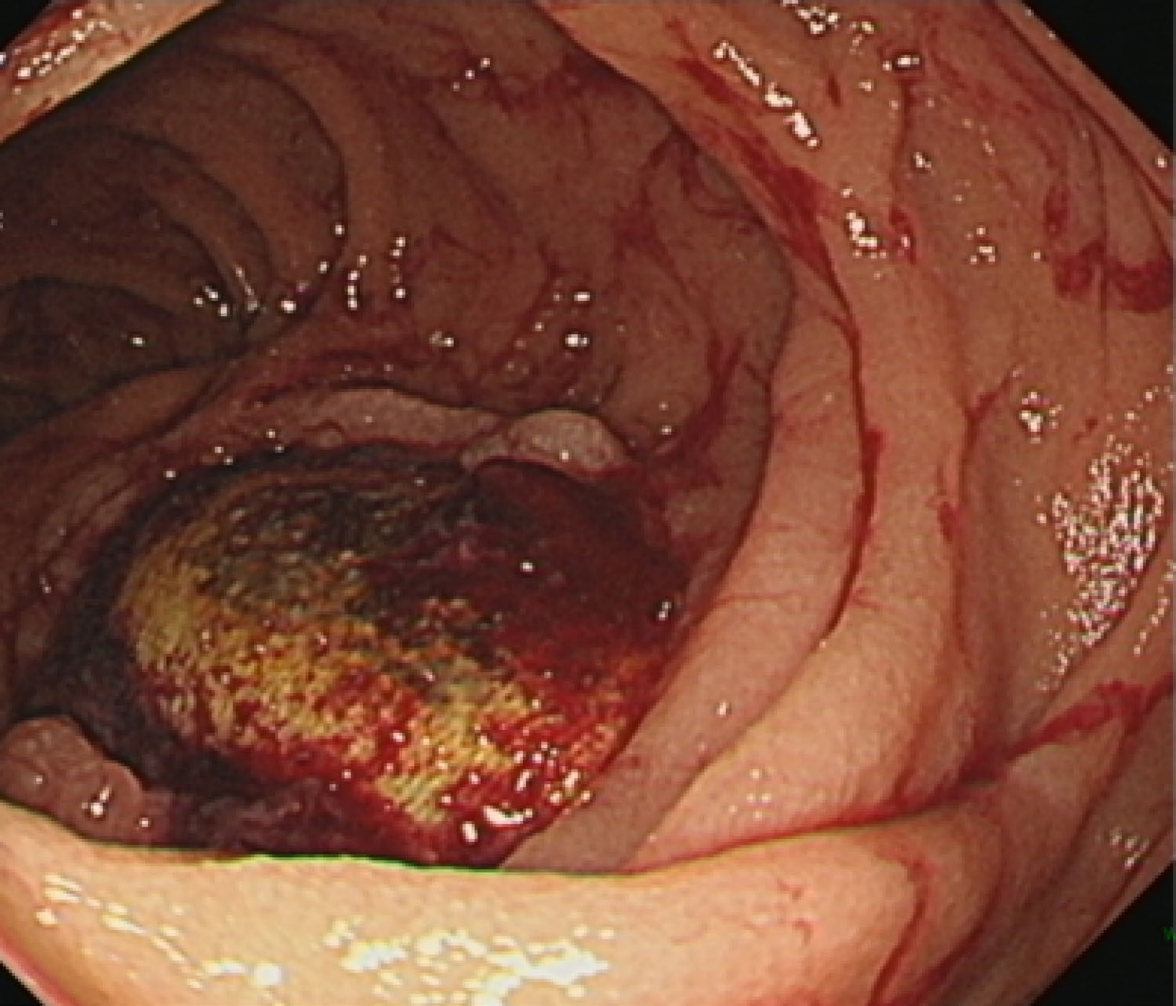
Esophagogastroduodenoscopy. The exposed prosthetic graft is visible in the descending duodenum

A 66-year-old woman had been diagnosed with Takayasu arteritis at the age of 15 years, and underwent subclavian–abdominal aortic bypass surgery for aortic stenosis at the age of 23 years, at our hospital. She reported lightheadedness for 1 month and was referred to another hospital for severe anemia. Plain computed tomography (CT) showed that the artificial blood vessel was close to the duodenum and compressing the descending duodenum (Fig. 1). Upper gastrointestinal endoscopy revealed an exposed artificial blood vessel in the descending duodenum (Fig. 2), and the patient was referred to our hospital for further examination and treatment.

**Fig. 3:**
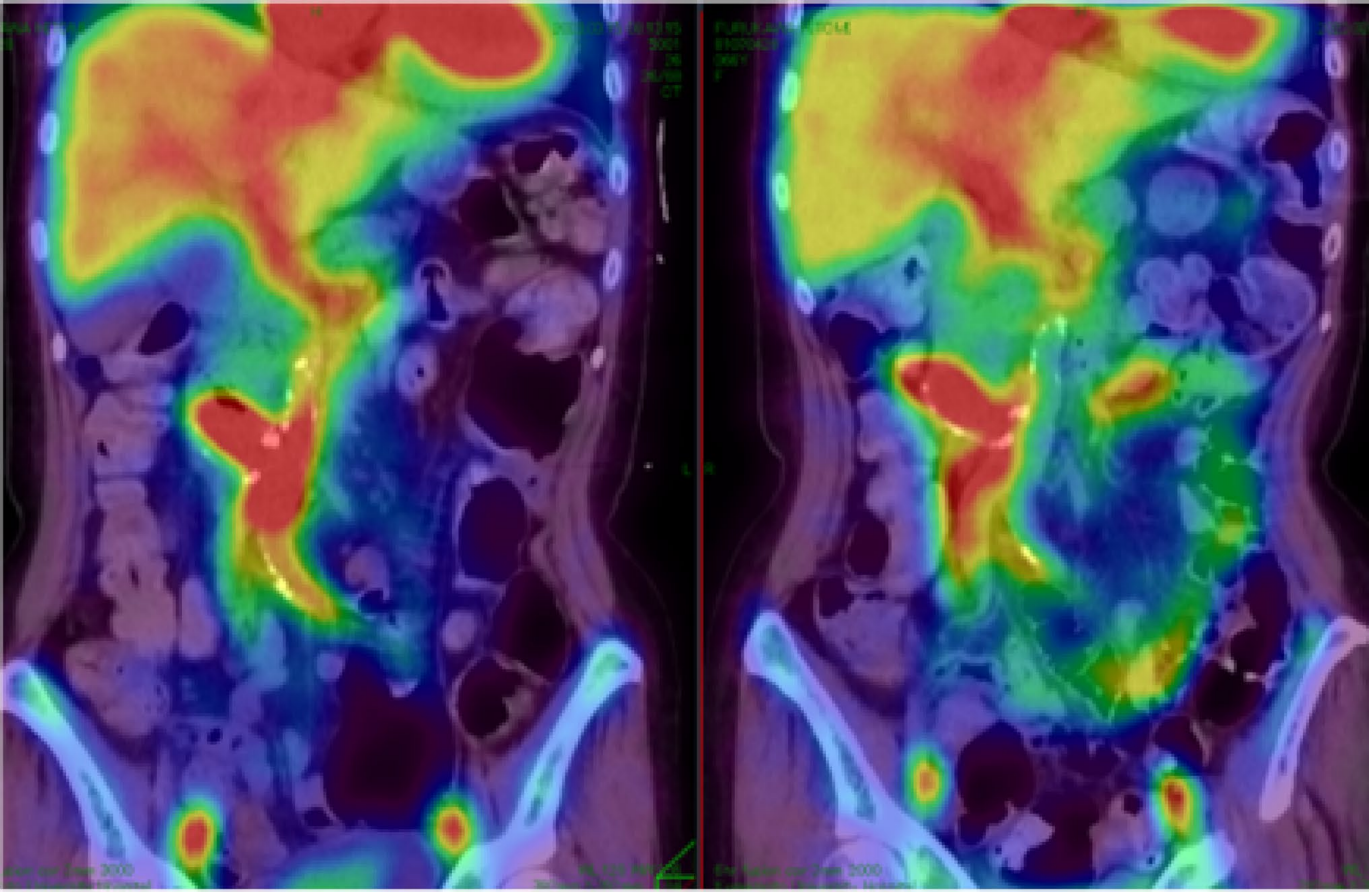
Bleeding scintigraphy. The images show bleeding in the jejunum that was suspected to originate from the duodenum. No obvious source of bleeding could be identified

Immediately after admission, the anemia improved following a blood transfusion. However, intestinal bleeding occurred, and the anemia again worsened. Angiography and endovascular treatment were attempted, but approaching the target vessels proved difficult because of aortic stenosis associated with Takayasu’s arteritis. Repeated bleeding scintigraphy (Fig. 3) failed to identify the location of active bleeding from the exposed part of the artificial blood vessel. However, we suspected that this was the source of the bleeding, and we chose surgery by a cardiovascular surgeon.

**Fig. 4:**
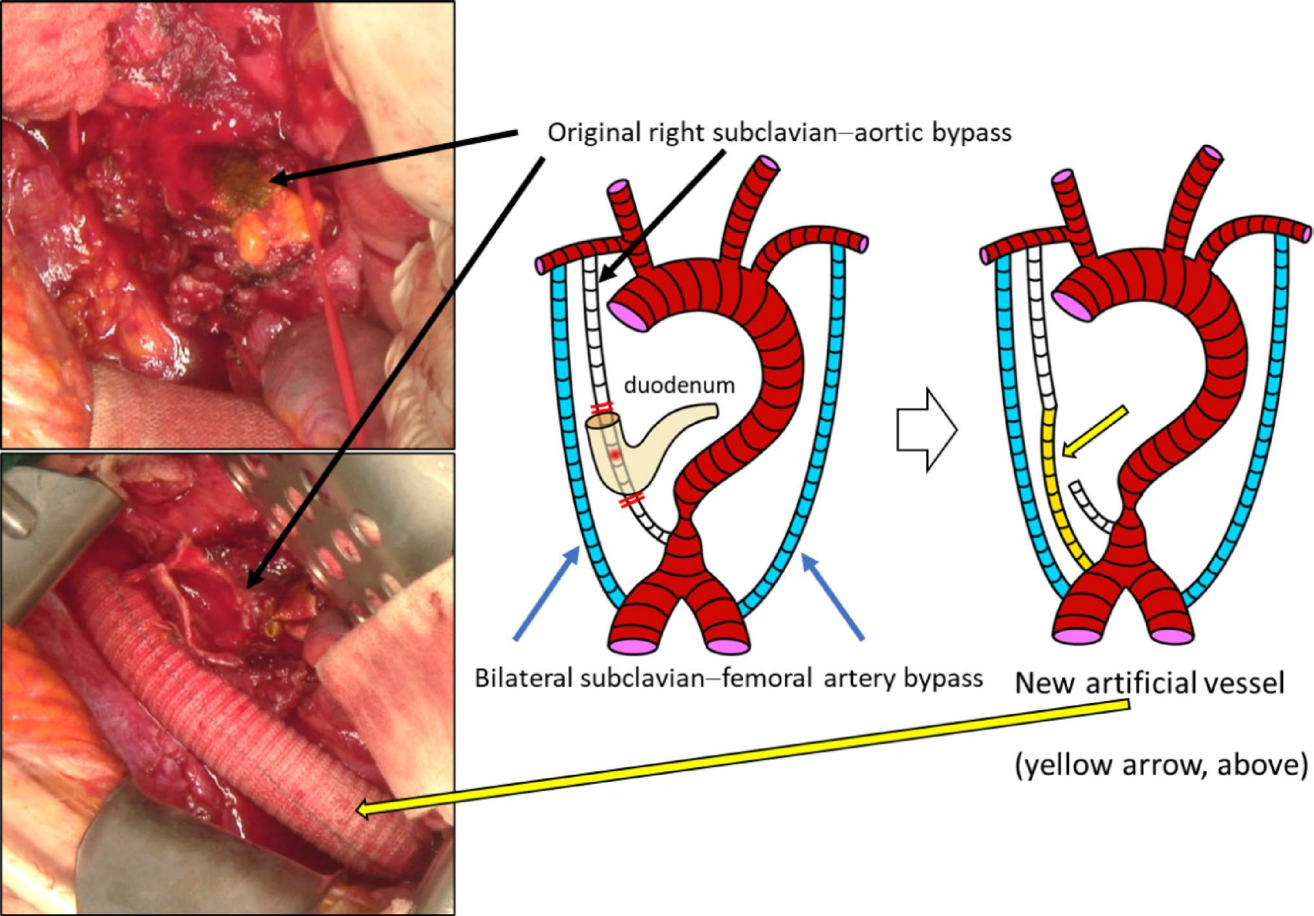
Intraoperative images and diagrams showing the revascularization surgery. The infected part of the artificial vessel was removed, and the peripheral side leading to the aorta was ligated. A new artificial vessel was then placed on the central side bypassed to the subclavian artery. The yellow arrow in the diagram on the right indicates the new artificial vessel

**Fig. 5:**
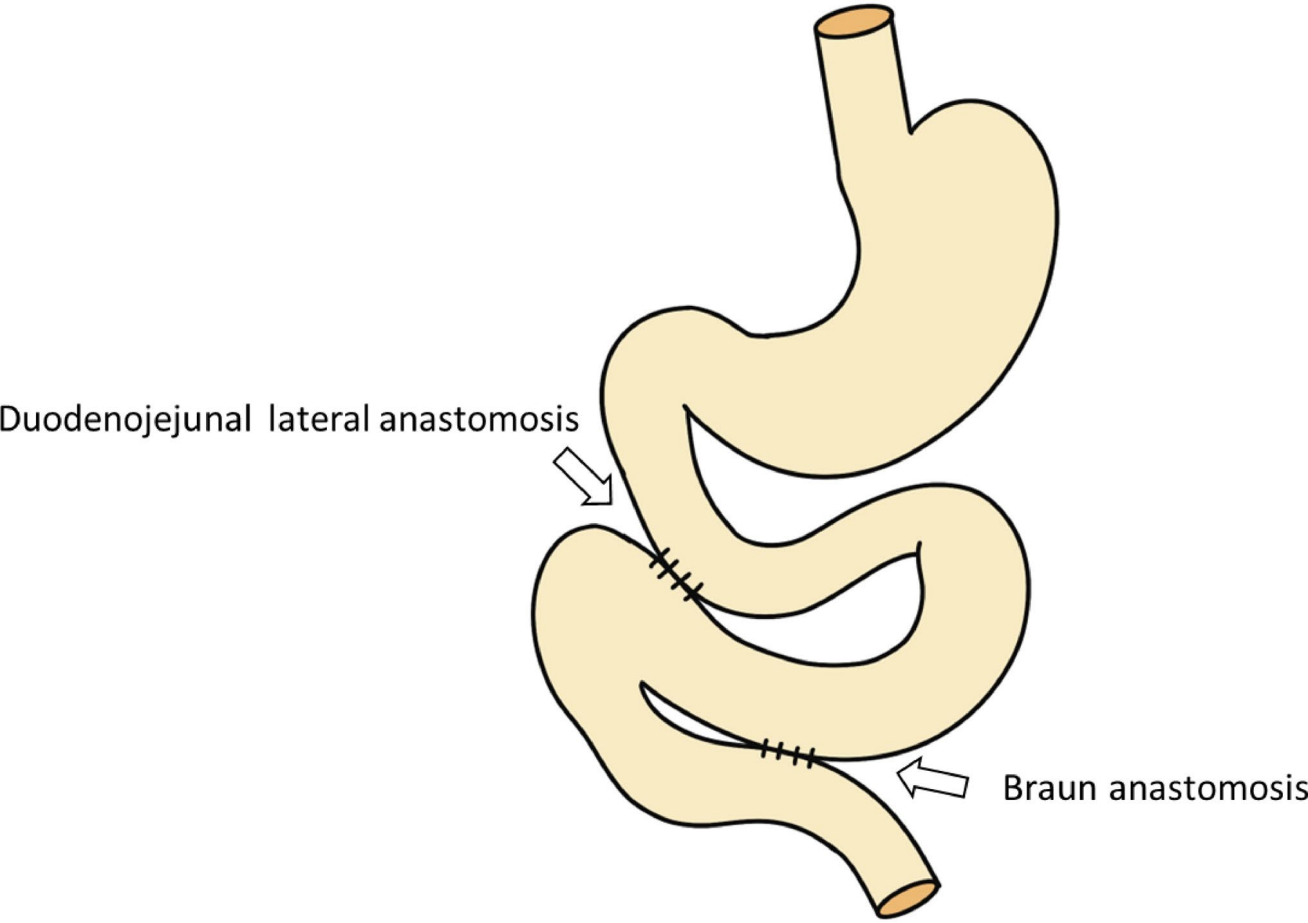
Diagram showing reconstruction of the digestive tract. Lateral anastomosis and Braun anastomosis were performed at the duodenal perforation site using an elevated intestine

Bilateral subclavian–femoral artery bypass was performed before the resection of the original artificial vessel. We then mobilized the right colon and performed the Kocher maneuver. With the aorta exposed, we taped the peripheral side of the artificial vessel. When the duodenum was removed from the artificial vessel, pulsatile bleeding was observed from a pinhole in the artificial vessel. During a clamp test performed in preparation for removing the old artificial vessel, a marked decrease in hepatic blood flow was observed; however, blood flow through the newly created bilateral subclavian–femoral artery bypass grafts remained good. Given the long interval since the initial bypass surgery, we considered that blood flow to the upper abdomen had come to depend on this collateral pathway. Therefore, the affected part of the artificial vessel was removed, the peripheral side leading to the aorta was sutured closed, a new artificial vessel was placed on the central side leading to the subclavian artery, and a bypass was added (Fig. 4). Blood pressure and blood flow improved after the bypass. After cessation of bleeding into the duodenum was confirmed, a partial wedge-shaped resection of the duodenum at the artificial vessel exposure site was performed. Lateral anastomosis and Braun anastomosis were then performed at the duodenal perforation site (Fig. 5). The operative time was 732 min, and the blood loss was 2639 ml.

There was no postoperative bleeding, and anticoagulation therapy began on postoperative day 3. Oral intake resumed on postoperative day 11.

## Discussion

Aortoenteric fistula is defined as a fistula between the aorta and the digestive tract, with primary aortoenteric fistula occurring as a complication of aortic aneurysm, infection, trauma, radiation therapy, and digestive ulcer [1]. SAEF, however, develops after artificial vessel placement [3]. The incidence of SAEF is higher in the abdominal aorta than in the thoracic aorta, and approximately one-third of cases involve fistula formation between the abdominal aorta and duodenum [4]. The cause of SAEF is considered to be pseudoaneurysm formation due to artificial vessel infection or hematoma formation, resulting in a fistula between the artificial vessel and the intestine. The causes of SAEF around the duodenum include the close anatomic relationship between the duodenum and the abdominal aorta, the loss of surrounding tissue during creation of the artificial vessel, and continuous mechanical stimulation from aortic pulsation and duodenal peristalsis [5]. The precise cause in our case is unclear. However, CT images showed compression of the duodenum by the artificial vessel, suggesting that mechanical stimulation may have contributed.

Typical symptoms of SAEF include anemia and gastrointestinal bleeding, but early definitive diagnosis is often difficult. In this case, we struggled to preoperatively identify bleeding from the exposed artificial vessel; early diagnosis and treatment are crucial with SAEF because aortic aneurysms may rupture soon after the disease’s onset, resulting in massive bleeding. CT is useful in diagnosis, direct confirmation of a fistula, and identifying the characteristic signs of SAEF: free air around the artificial graft and inflammation and thickening of the aorta. Upper gastrointestinal endoscopy is recommended for diagnosis to exclude other causes of gastrointestinal bleeding. Findings that positively suggest SAEF on upper gastrointestinal endoscopy include exposed grafts, surrounding bleeding, and ulcers, but the diagnostic rates vary [6]. Small intestinal endoscopy and colonoscopy may also be used to thoroughly observe the duodenum and jejunum [6]. In a previous report, lower gastrointestinal endoscopy was performed for exclusion diagnosis [6].

There is no established treatment for SAEF. Previous reports have carried out endovascular aortic repair for hemostasis in the acute phase and in two-stage radical surgery (Table 1) [2, 7–12]. Multiple reports have confirmed the effectiveness of endovascular aortic repair as an initial treatment for avoiding infection and quickly stabilizing hemodynamics, with no significant difference in postoperative mortality compared with graft resection or primary aortic repair [1]. Another report showed that the 2-year survival rate was significantly better after endovascular treatment than after open surgery [13]. However, in our case, endovascular treatment was difficult because of Takayasu’s arteritis. The effectiveness of endovascular approaches may be limited in the presence of vascular stenosis or deformation. Although radical surgery with total graft removal and revascularization has been performed, the prognosis was poor because of complications such as bypass occlusion and anastomotic leakage [2]. Recently, partial replacement surgery using artificial blood vessels and bypass has become common. This approach is performed when total replacement is difficult, such as with difficulty exposing the entire artificial blood vessel [2]. Although there are no clear criteria for the timing of surgery, with signs of infection, surgery should be performed after infection is controlled with antibiotic therapy [8].

**Table 1:** Previous reports of SAEF

Case	Age (years)	Sex	Chief Complaint	Medical History	Surgical History	Time Since the Primary Surgery (years)	Pathology	Initial Intervention	Days to Definitive Surgery	Detected Bacteria/Fungi	Antibiotics/Antifungals	Complications	Hospital Stay (days)	Outcome
1	66	Female	Light-headed	Takayasu arteritis	Subclavian−abdominal aortic bypass	43	AEF (Duodenum)	Arterial bypass, Partial duodenectomy	32	Not identified	CMZ	None	23	Survived
2	63	Male	Anemia, abdominal pain	signet ring cell carcinoma	Open AAA repair	1.2	AEF (duodenum, jejunum)	graft replacement	10	Not specified	Not specified	None	-	Survived
3	36	Male	Melena, abdominal pain	Iliac artery occlusive disease	Aorto-femoral/iliac bypass	7	AEF (third part of duodenum)	Axillary-femoral bypass, Duodenojejunostomy	0	Not specified	Not specified	None	7	Survived
4	63	Male	Hematochezia, hematemesis	None	Infrarenal AAA prosthetic repair	8	AEF (duodenum)	Thoracoabdominal replacement, duodenal closure	0	Not specified	Not specified	None	-	Survived
5	76	Male	Hematemesis	Hypertension	Juxtarenal AAA replacement	6.5	AEF (Duodenum)	Partial graft replacement, Partial duodenectomy	0	Streptococcus anginosus	TAZ/PIPC, AMPC	Cholecystitis, Duodenal suture insufficiency	59	Survived
6	75	Male	Hematemesis	Hypertension, Dyslipidemia, Diabetes mellitus, CKD, Angina Pectoris	Infrarenal AAA replacement	2.8	AEF (Duodenum)	EVAR	86	Granulicatella adiacens, Staph epidermidis (MRS)	MEPM, VCM	Graft infection	150	Died (Sepsis)
7	70	Male	Hematemesis	Hypertension, Dyslipidemia, Angina Pectoris	Infrarenal AAA replacement	5	AEF (Duodenum)	EVAR	32	Staphylococcus aureus (MRSA)	-	None	110	Survived
8	70	Male	Fever	Hypertension, Sick sinus syndrome(post-PCI)	Infrarenal AAA replacement	3.3	AEF (Duodenum)	Partial graft replacement with rifampicin-soaked graft	35	Staphylococcus epidermidis (MRS), Leuconostoc	TAZ/PIPC, MEPM	None	82	Survived
9	75	Female	Anorexia	infectious endocarditis, Cerebral Infarction, atrial fibrillation, Hypertension, hyperuricemia	Infrarenal mycotic AAA replacement	1.5	AEF (Duodenum)	Partial graft replacement with rifampicin-soaked graft	20	Olsenella uli, Slackia exigua, Atopobium parvulum	MEPM, VCM	Intramuscular abscess	35	Survived
10	67	Male	Right thigh swelling, fever	Hypertension, coronary angioplasty, Leriche syndrome	Aortic-bifemoral bypass	6	AEF (Duodenum)	Prosthetic explantation, bowel resection	0	E. coli, Klebsiella, Enterococcus	Imipenem, Teicoplanin, Amikacin	Necrotizing fasciitis	1	Died (Multiple organ failure)
11	68	Male	Massive hematemesis	Ischemic heart disease, Hyperlipidemia	Infrarenal aortic ligation, Axillobifemoral bypass	7	Recurrent AEF	Percutaneous transarterial embolization	8	Enterococcus faecalis	Not specified	None	-	Survived
12	63	Male	Anemia, abdominal pain	signet ring cell carcinoma	Open AAA repair	1.2	AEF (duodenum, jejunum)	graft replacement	10	Not specified	Not specified	None	-	Survived

The table shows a summary of case reports on SAEF in the English literature that were identified in a PubMed search from November 2022. Our case is number one on the list

SAEF, secondary aortoenteric fistula; AAA, abdominal aortic aneurysm; CKD, chronic kidney disease; PCI, percutaneous coronary intervention; EVAR, endovascular aortic repair; MRS, methicillin-resistant; MRSA, methicillin-resistant Staphylococcus aureus; CMZ, cefmetazole; TAZ, tazobactam; PIPC, piperacillin; AMPC, ampicillin; MEPM, meropenem; VCM, vancomycin

The interval between the initial surgery and the onset of SAEF has been reported to range from as short as 2 days to as long as 23 years [14]. In our case, the interval was even longer than previously described. Additionally, endovascular treatment was difficult owing to arteritis. Therefore, we initially performed radical surgery comprising partial artificial vessel resection, local duodenal resection, and reconstruction. Bleeding has not recurred at the time of this report, and the patient’s symptoms improved with minimal intestinal reconstruction. 

SAEF is difficult to diagnose and should be considered in patients with anemia or gastrointestinal bleeding and a history of artificial vascular replacement surgery. Even with a definitive diagnosis, choosing the best treatment procedure is difficult. In this case, the diagnosis was made by endoscopy, and the patient was saved by revascularization and minimal intestinal reconstruction. 

## Abbreviations

SAEF: Secondary aortoenteric fistula
CT: Computed tomography
